# Measuring strategies for learning regulation in medical education: Scale reliability and dimensionality in a Swedish sample

**DOI:** 10.1186/1472-6920-12-76

**Published:** 2012-08-15

**Authors:** Samuel Edelbring

**Affiliations:** 1 Centre for Medical Education, Department LIME, Karolinska Institutet, Berzelius väg 3, 171 77 Stockholm, Sweden

**Keywords:** Regulation strategies, Self-regulated learning, Approaches to studying, Validation

## Abstract

**Background:**

The degree of learners’ self-regulated learning and dependence on external regulation influence learning processes in higher education. These regulation strategies are commonly measured by questionnaires developed in other settings than in which they are being used, thereby requiring renewed validation. The aim of this study was to psychometrically evaluate the learning regulation strategy scales from the Inventory of Learning Styles with Swedish medical students (*N* = 206).

**Methods:**

The regulation scales were evaluated regarding their reliability, scale dimensionality and interrelations. The primary evaluation focused on dimensionality and was performed with Mokken scale analysis. To assist future scale refinement, additional item analysis, such as item-to-scale correlations, was performed.

**Results:**

Scale scores in the Swedish sample displayed good reliability in relation to published results: Cronbach’s alpha: 0.82, 0.72, and 0.65 for *self-regulation*, *external regulation* and *lack of regulation* scales respectively. The dimensionalities in scales were adequate for *self-regulation* and its subscales, whereas *external regulation* and *lack of regulation* displayed less unidimensionality. The established theoretical scales were largely replicated in the exploratory analysis. The item analysis identified two items that contributed little to their respective scales.

**Discussion:**

The results indicate that these scales have an adequate capacity for detecting the three theoretically proposed learning regulation strategies in the medical education sample. Further construct validity should be sought by interpreting scale scores in relation to specific learning activities. Using established scales for measuring students’ regulation strategies enables a broad empirical base for increasing knowledge on regulation strategies in relation to different disciplinary settings and contributes to theoretical development.

## Background

Students’ independent learning in terms of monitoring and guiding their own learning process is considered fundamental for students’ achievements in higher education [1,2]. With roots in adult learning [3], the concept of self-regulated learning has developed in social cognitive theory [4] and psychology of learning [5]. Self-regulated learning is considered to be a capability that comprises aspects of responsibility for one’s own learning, such as evaluating, regulating, and setting goals for the learning process [1,5]. Theorists describe students’ learning regulation strategies as indirectly influencing the process of learning, thereby influencing learning outcomes [5]. The concept has been found to engage students, educators as well as policy makers [6]. Inspired by incentives for lifelong learning, and student centred education, the large interest for self-regulated learning in higher education has also reached medical education [7,8].

The theoretical constructs of regulation strategies are commonly measured by questionnaire scales for quantification of results. The issue of validity—the degree to which the scale measures what it purports to measure, is of central concern in this practice. Messick [9] stated that validity is a characteristic of the interpretation of results and does not pertain to the instrument itself. Cultural differences, changes in student characteristics over time, varying teaching and study practices between disciplines and national educational systems require renewed assessment and fine tuning of instruments [10]. Hence, whether or not established questionnaires function in new environments is an empirical question, the results need to undergo psychometric evaluation for the population in question [11].

The predominant method for evaluating scale quality is to measure the internal scale consistency with Cronbach’s alpha, which functions as a lower bound of reliability [12]. A more thorough analysis would also look into the dimensionality of the item set—the extent to which scale items relate to one construct—thereby allowing using a sum score to accurately indicate levels of the latent trait. Because it has been shown that high Cronbach’s alpha does not guarantee unidimensionality, additional measures are needed to assess whether items comprise a unidimensional scale regarding the construct it is intended to cover [13].

A fairly recent scale analysis method is the Mokken scale analysis [14,15]. This method presents an alternative to factor analysis and is categorised under the non-parametric item response theory (IRT). Mokken analyses evaluate scale dimensionality based on Loevinger’s definition of homogeneity [16]. In addition to dimensionality, Mokken scale analysis also has methods for testing the IRT models of monotone homogeneity and double monotonicity [17]. Monotone homogeneity means that respondents can be ordered on a latent trait using scale scores. The more restrictive model of double monotonicity means that the scale items also can be hierarchically ordered in relation to the latent trait. Furthermore, the Mokken method makes fewer assumptions on empirical data than does traditional factor analysis (e.g. regarding models and linearity of the item-construct relation) [18-20].

The Inventory of Learning Styles (ILS) [5] was developed in a European setting specifically for the higher education context and has been widely used and reviewed in researching students’ learning patterns [2,21,22]. In addition to regulation strategies the complete ILS covers processing strategies, mental models of learning, and learning orientations. The regulation scales purport to measure how students usually approach studying in terms of regulation strategies. The three main scales—*self-regulationexternal regulation,* and *lack of regulation*—are represented by 28 items. *Self-regulation* contains the subscale dimensions *learning process and result* and *learning content,* which are concerned with the degree to which students plan processing activities, diagnose the cause of learning problems that occur, and direct themselves toward learning objectives of their own. *External regulation* contains the subscales *learning results* and *learning processes*. *External regulation* concerns the degree to which students rely on didactic aids, such as formal learning objectives and assignments. *Lack of regulation* concerns the inability to regulate one’s own learning and perceived lack of external support [5].

Increased knowledge of regulation strategies in student groups contributes both to direct student benefit and to research purposes. Regulation strategy scales have been used as a self-diagnostic tool for stimulating reflection on learning processes and raising awareness about study strategies among teachers and students [2,23,24]. Research on regulation strategies contributes with knowledge regarding the type of guidance students need from the teacher and the course structure, both in larger structures, such as comparisons between traditional and problem-based settings [25], and in relation to specific course activities [26]. This knowledge forms an important base for making full use of students’ motivation to learn and adapting corresponding course design and teacher guidance [27]. Differences in how self-regulated learning is enacted and its consequences for learning highlight the need for investigating these attributes in relation to each discipline [28]. In the field of medical education, concern has been voiced that much of previous efforts to enhance learners’ autonomy have been based on loose assumptions rather than systematic research [29]. The rich body of empirical and theoretical literature from other disciplines cannot replace contextualised empirical findings in medicaleducation [8]. The aim of this study was to psychometrically evaluate a translation of established regulation strategy scales in a Swedish medical education context.

## Methods

### Participants

The sample consisted of medical students participating in a clinical clerkship preparatory course. The students were approached cross-sectionally during the course, in four hospitals affiliated with the medical university of Karolinska Institutet, Sweden. A pilot was conducted in two courses in autumn 2008 (*n* = 44, response rate = 67 %) followed by a cross-sectional study in all four courses in spring 2009 (*n* = 206, response rate = 83 %). At this point, the students were at the verge of entering the more clinical oriented phase of the medical programme.

### Materials and procedure

The regulation strategy scales from the 120-item version of the Inventory of Learning Styles (ILS) [5] were translated, piloted, revised, and used in 2008–2009. The 28 items were composed of Likert-type statements, with response alternatives denoting the frequency with which students use the proposed activity: e.g. ‘I add something to the subject matter from other sources’, ‘I do this seldom or never’, ‘I do this sometimes’, ‘I do this regularly’, ‘I do this often’, ‘I do this almost always’ (the answer categories are represented by the numbers 1–5).

The questionnaire was first translated by the author to Swedish. Item wordings were then examined in relation to the Norwegian translation [25] as the two languages are closely related, and then reviewed by two bilingual medical educationalists regarding content validity. After the pilot, item wordings were further refined through telephone interviews and a group interview, with three and five respondents, respectively.

Incomplete responses were approached differently when analysing the data from the pilot and the revised version. In the pilot, non-systematic missing values were approached with “hot deck imputation” —copying values from other respondents with similar but complete response patterns in the scale analysis in order to make full use of the small sample [30]. In the revised setting incomplete responses (10) were discarded from the scale analysis. The original item order from the ILS was retained, although item numbering differs due to the standalone regulation strategy version being shorter. The study has been ethically reviewed by the local ethical board (rn: 2008/822-31/5).

### Validation criteria

Internal reliability for the scales was estimated with Cronbach’s alpha. Scale dimensionality was estimated by Mokken scale analysis based on Loevinger’s coefficient *H *[16]. *H* > 0.3 means that items have enough in common to trust the ordering of persons by using the scale scores [17]. The common practise for interpreting dimensionality by means of coefficient *H* is that a scale is considered weak when the *H*-estimate is between 0.3 and 0.4, medium when between 0.4 and 0.5 and strong when > 0.5. All item pairs within each scale were further investigated regarding covariance (*Hij*), and individual item relation to the scale (*Hi*) [14]. The Mokken automated scale partitioning was used to investigate whether the established factor structure would be replicated. Interrelations between scales were calculated by Spearman rank order correlation on summed scale scores due to its ordered data origin. Invariant item ordering is one method used to check for double monotonicity, i.e. the extent to which items can be ordered hierarchically in the scale in relation to the regulation strategy construct. This aspect was estimated by coefficient *HT*. Proposed interpretations of this coefficient indicate that *HT* < 0.3 means that the item ordering is inaccurate regarding item ordering; low accuracy between 0.3 and 0.4, medium when between 0.4 and 0.5, and high accuracy when > 0.5 [31]. Statistical analyses were performed in the statistical package R version 2.13.0 [32]. The R Mokken library version 2.5 was used for Mokken scale analysis.

## Results

### Pilot

Reliability, as measured by Cronbach’s alpha, ranged from 0.60 to 0.80 in the three main scales (Table 1). Mokken dimensionality analysis showed that the two self-regulation subscales were of adequate dimensionality (*H* > 0.3) (Table 2). The item pair covariance (*Hij*) ranged from −0.21 to 0.77 and individual item dimensionality (*Hi*) ranged from 0.07 to 0.46 (Table 3). Despite some negative *Hij* values, items generally contributed to their respective dimensions.

**Table 1:** Internal consistency of regulation scales

**Regulation scales and subscales**	**No. items**	**Cronbach’s alpha**	
**Main scales**		**Pilot**	**Revised**
Self-regulation	11	0.80	0.82
External regulation	11	0.64	0.72
Lack of regulation	6	0.60	0.65
**Subscales**			
Self-regulation of learning content	4	0.65	0.66
Self-regulation of process and results	7	0.75	0.81
External regulation of learning results	5	0.54	0.57
External regulation of learning processes	6	0.47	0.67

**Table 2:** Mean scores of the CCOG items for the first and second interview *

**Item**	**Pre**	**Post**	**Difference**	
	**mean**	**mean**	**mean**	**(95% CI)**^**†**^
**Section: Initiating the Session**	2.9	2.6	0.3	(0.1 - 0.5)
Greets patient	2.0	1.8	0.2	(−0.1 - 0.5)
Introduces self and role	2.9	2.6	0.3	(−0.2 - 0.9)
Demonstrates respect	2.0	2.0	0.0	(−0.2 - 0.3)
Identifies and confirms problems list	3.1	2.5	0.6	(0.2 - 1.1)
Negotiates agenda	4.2	4.1	0.1	(−0.4 - 0.6)
**Section: Gathering Information**	2.8	2.3	0.5	(0.2 - 0.9)
Encourages patient to tell story	3.0	2.2	0.7	(0.3 - 1.2)
Appropriately moves from open to closed questions	3.1	2.4	0.7	(0.2 - 1.1)
Listens attentively	2.3	1.9	0.4	(0.1 - 0.8)
Facilitates patient′s responses verbally and non-verbally	2.6	2.1	0.5	(0.1 - 0.9)
Uses easily understood questions and comments	2.3	2.1	0.2	(−0.2 - 0.6)
Clarifies patient′s statements	2.8	2.5	0.3	(−0.1 - 0.7)
Establishes dates	3.5	2.5	1.0	(0.5 - 1.5)
**Section: Understanding Patient′s Perspective**	3.4	2.8	0.5	(0.2 - 0.9)
Determines and acknowledges patient′s ideas re: cause	3.7	3.4	0.3	(−0.2 - 0.9)
Explores patient′s concerns re: problem	3.1	2.5	0.6	(0.2 - 1.0)
Encourages expression of emotions	3.1	2.8	0.3	(−0.1 - 0.7)
Picks up/responds to verbal and non-verbal clues	3.4	2.8	0.6	(0.2 - 0.9)
**Section: Providing Structure to Consultation**	3.3	2.9	0.4	(0.1 - 0.8)
Summarises at end of a specific line of inquiry	3.9	3.4	0.5	(−0.1 - 1.0)
Progresses using transitional statements	3.3	2.9	0.4	(−0.1 - 0.9)
Structures logical sequence	3.3	2.8	0.5	(0.1 - 1.0)
Attends to timing	2.8	2.5	0.3	(−0.2 - 0.7)
**Section: Building Relationship**	2.7	2.3	0.4	(0.1 - 0.8)
Demonstrates appropriate non-verbal behaviour	2.6	2.2	0.5	(−0.1 - 0.9)
If reads or writes, doesn′t interfere with dialogue/rapport	2.7	2.3	0.4	(0.0 - 0.8)
Is not judgemental	2.4	2.1	0.3	(−0.1 - 0.8)
Empathises with and supports patient	2.9	2.4	0.5	(0.1 - 1.0)
Appears confident	3.1	2.5	0.6	(0.1 - 1.1)
**Section: Closing the Session**	3.7	2.7	1.0	(0.6 - 1.4)
Encourages patient to discuss any additional points	4.0	2.9	1.1	(0.6 - 1.6)
Closes interview by summarising briefly	3.9	3.0	0.9	(0.4 - 1.4)
Contracts with patient re next steps	2.9	2.0	0.9	(0.4 - 1.5)
**Total score**	3.1	2.6	0.5	(0.2 - 0.8)

* 5-point scale of all items; pre and post (scale: 1 = excellent, 5= deficient). † 95% confidence interval.

**Table 3:** **Individual item dimensionality (*Hi *) in relation to scales**

**Self-regulation**	**Item**	**7**	**13**	**21**	**27**	**10**	**11**	**15**	**17**	**23**	**25**	**26**
	*Hi*	0.27	0.28	0.29	0.29	0.30	0.26	0.27	0.38	0.41	0.40	0.37
External regulation	Item	16	4	24	2	5	8	1	14	19	28	22
	*Hi*	0.30	0.27	0.27	0.22	0.22	0.19	0.18	0.18	0.18	0.14	0.11
Lack of regulation	Item	3	9	20	12	6	18					
	*Hi*	0.34	0.29	0.26	0.24	0.23	0.20					
Self-regulation of learning content	Item	7	21	27	13							
	*Hi*	0.41	0.41	0.37	0.31							
Self-regulation of process and results	Item	25	17	23	10	26	11	15				
	*Hi*	0.50	0.48	0.45	0.41	0.35	0.34	0.31				
External regulation of learning results	Item	14	4	5	28	22						
	*Hi*	0.28	0.24	0.24	0.23	0.15						
External regulation of learning processes	Item	16	24	2	8	19	1					
	*Hi*	0.36	0.30	0.28	0.27	0.25	0.20					

Exploratory scale partition resulted in three Mokken scales in the pilot (Table 4). The first scale only included items from the established *self-regulation* scale. Mokken scale 2 corresponded to the *external regulation* scale, and scale 6 to *lack of regulation*. Dimensionality coefficient *H* ranged from 0.15 to 0.36 (Table 2). Data from student interviews indicated that the items required a lot of thought, and that the students perceived several items as being similar to each other.

**Table 4:** Resulting scales from Mokken exploratory partitioning

**Pilot version**		**Revised version**	
**Item no.**	**Item *H***	**Item no.**	**Item *H***
Scale 1			
23	0.77	25	0.67
17	0.77	10	0.67
26	0.65	17	0.63
21	0.57	23	0.57
7	0.51	26	0.50
27	0.47	22	0.46
10	0.42	11	0.42
Scale 2		Scale 2	
24	0.64	24	0.54
16	0.64	4	0.54
4	0.55	16	0.48
Scale 3		8	0.42
18	0.63	Scale 3	
12	0.63	21	0.53
Items not assigned to scales: 1,2,3,5,6,8,9,11,13,14,15,19,20,22,25,28		7	0.53
		27	0.44
		13	0.38
		Scale 4	
		9	0.51
		3	0.51
		6	0.41
		Scale 5	
		28	0.41
		14	0.41
		Scale 6	
		18	0.33
		12	0.33
		Items not assigned to scales: 1,2,5,15,19,20	

### Revised version

Based on student interviews, the wordings of the items were refined slightly, after which the questionnaire was distributed to the cohort of medical students taking a clinical clerkship preparatory course at four teaching hospitals. The reliability for main scales ranged from Cronbach’s alpha 0.65 to 0.82 (Table 1).

Evaluation of the established scales showed self-regulation and its subscales to be of adequate (*H* > 0.3) dimensionality (Table 2). The individual item dimensionality (*Hi*) ranged from 0.11 to 0.50, with two items (22 and 28) displaying low, but positive, scale dimensionality (Table 2). Pair-wise covariance (*Hij*) ranged from −0.09 to 0.67. All item pairs, except three, displayed positive *Hij* values. Negative *Hij* pairs were 8–14 and 8–28 in scale *external regulation* and 6–18 in *Lack of regulation*, ranging from −0.01 to −0.09.

Six Mokken scales were extracted from the responses and compared to the theoretical factor structure (Table 3). The first one included six out of seven items from *self-regulation of learning process and results* and one (item 22) from the *external regulation* scales. Mokken scale 2 included three items from *external regulation of learning processes* and one from *external regulation of learning results*. The third scale was identical to the *self-regulation of learning content.* The fourth and sixth scales included three and two items, respectively, from *lack of regulation*. The fifth scale contains two items from *external regulation of learning results*.

No internal correlation was found between main scales (*Rho* ≤ 0.14 in the pilot and ≤ 0.08 in the revised setting). As expected, subscales correlated significantly with each other (*p* < 0.01) for *self-regulation* (*Rho* = 0.47) and *external regulation* (*Rho* = 0.39). Three scales (*external regulation, sub scale self-regulation of learning content,* and *external regulation of learning processes*) displayed invariant item ordering coefficients >= 0.3 (Table 5).

**Table 5:** Invariant Item Order (IIO) scale assessment

**Scales**	***HT***
Self-regulation	0.23
External regulation	0.30
Lack of regulation	0.16
Self-regulation of learning content (subscale)	0.53
Self-regulation of process and result (subscale)	0.13
External regulation of learning results (subscale)	0.40
External regulation of learning processes (subscale)	0.18

## Discussion

Adequate dimensionality, divergent validity, and scale consistency contribute validity to the *self-regulation (SR)* scale and its subscales in the medical education setting. However, the analyses indicate that some of the items do not contribute optimally in the regulation scales and that *external regulation (ER)* and *lack of regulation (LR)* scales were weak regarding dimensionality in this sample.

Because psychometrics is about measuring the unobservable, there is not one single criterion against which we can assert fulfilment of the goal of trustworthy tools. Therefore, several aspects, taken together, form a basis for assessment and basis for further scale development. The internal consistency in this sample was very good in comparison with other studies using the scales. Cronbach’s alpha values for the three main scales (*SR*: 0.82, *ER*: 0.72, and *LR*: 0.65) were exceeding those obtained in a Norwegian medical setting (*SR*: 0.73, *ER*: 0.69, and *LR*: 0.57) [25] and generally exceeded previously reported *Cronbach’s alpha* of 0.48 to 0.81 in several studies in the Netherlands [22], 0.46 to 0.72 in British settings [2], and 0.69 to 0.75 in a Finnish pharmacy setting [33].

Dimensionality, as assessed by Mokken scale analysis, was higher in the dataset from the revised version, although the estimates were not ideal (Table 2). The strongest regulation scale was the subscale *Self-regulation process and results,* with coefficient *H* = 0.41. Following empirically derived rules of thumb of *H*-interpretation, this falls into the category of moderate dimensionality [17]. Of the main scales, *self-regulation* got highest value with *H* = 0.32 which means it is a weak scale regarding dimensionality. *External regulation* scale displayed lowest dimensionality (*H* = 0.21). This finding corresponds with this dimension not being detected at all in a Finnish sample [26]. Higher *H* coefficient values for sub scales are explained by their items being more narrowly connected to each other, whereas the main scales approach the strategy dimensions more broadly. The implication for scales with low dimensionality is that ranking of participants with scores in a narrow range will be less accurate.

On a more detailed level, the *Hi* estimate contributed with insight into how single items contribute to scale dimensionality (Table 2). In general, all items contributed to their respective scale, except items 22 and 28, which did not seem to contribute much to the scale (*external regulation*). The characteristic of item 22 is discussed below. Item 28 concerns fulfilment of assignments during the course which makes it less appropriate for a clinically (practically) oriented course. The total dimensionality coefficient (*H*) for that scale would increase removing these two items. However, that would remove nuances from the scale construct, and hence, from the theoretical base. Since both items score positively on the *Hi*, they contribute, however little, to the scale construct. The practical implications are that the scale is less accurate in distinguishing between respondents with similar scale scores than if all items contribute highly to the scale construct. When disregarding previous partitioning and exposing all items to exploratory scale partitioning, they risk being regrouped in ways that were not initially intended. However, the theoretical scale structure was broadly replicated in the data. All aspects of the established scales were represented in the six Mokken scales. The largest group is Mokken scale 1, with six of the seven *self-regulation of learning process and results* items represented. Scale 2 contains items from *external regulation*, mostly from *external regulation of learning process.* Scale 3 was identical to *self-regulation of learning content.* Scale 5 has two items from *external regulation of learning results,* and scales 4 and 6 correspond with *lack of regulation.*

With the exception of one item (22), the six exploratory Mokken scales correspond well with the theoretical partitioning established in previous research (Table 3). However, in this sample, item 22 converges with *self-regulation of learning process and results,* although it belonged to *external regulation of learning results* in the established partitioning. This item also displayed low item-scale homogeneity (*Hi)*. The item concerns the frequency with which students thoroughly apply themselves to the methods dealt with in a course. Considering the context in which respondents were situated—the clinical preparatory course—the “methods” could be interpreted as diagnostic methods, and thus a core aspect of the course. Training to apply these methods could fit well into the scope of a self-regulated goal. Consequently, this item does not function well in discriminating between self- and externally regulated strategies, but contributes to both constructs in this setting.

Regulation strategies as such are theorised as not only relating to individual preference, but also to the specific learning situation [5,34,35]. Consequently, the situative aspect should be considered in data collection and interpretation. Comparisons between different overarching curricula, such as PBL and traditional ones, should expect variations in how the construct is interpreted [25,36]. Comparisons within the same educational culture and setting, contribute to establish the construct validity of regulation strategies as measured by these scales. When comparing this study’s scale means to other traditional Scandinavian curricula, a similar distribution of pattern is discerned, dominated by *external regulation,* followed by *self-regulation* and *lack of regulation* (Table 6).

**Table 6:** Regulation strategy mean scores from the Swedish setting compared with Norwegian medical education settings, using problem-based (PBL) and traditional curriculum

	**Sweden, Medicine (current study)**	**Norway, Medicine, PBL**	**Norway Medicine, trad.**
Self-regulation	2.72	2.88	2.45*
External regulation	3.06	2.71	2.67*
Lack of regulation	2.55	2.51	2.32

Mean values from (Hofgaard Lycke, et al., 2006; Stromso, Grottum, & Hofgaard Lycke, 2004). * Calculated from subscale means in (Hofgaard Lycke et al., 2006).

As the three regulation strategies are different constructs, they are not expected to interrelate; i.e. they should display divergent validity. Nevertheless, the subscales of the *self-* and *external regulation* scales should relate, to some extent, although covering different facets. The non-interrelation identified among the main scales contributes indirectly to their divergent validity. Interrelations between subscales were significant, although somewhat weaker than those reported elsewhere [2]. Judging by their face value, one could assume that *self-regulation* and *external regulation* would be each other’s opposites and, hence, correlate negatively; however, in this sample, they did not. Research shows that students can successfully combine the two regulation strategies, suggesting a dynamic relationship where external regulation has modelling and scaffolding functions [23].

Invariant item ordering (IIO) is not a claimed property of the ILS’s regulation strategy scales. Scale items contribute within the regulation strategy scales, even if not ordered in a hierarchy. Nevertheless, the test for IIO contributed additional validation data in disclosing to what extent respondents conceive the items in a similar manner. Three scales displayed adequate IIO accuracy, while the overall low IIO estimates for other scales express variation in how respondents approach the scale items (Table 5).

### Implications and further development

The gains of having an established instrument that can be used in different disciplines and in different cultural settings should be considered when choosing and assessing methods to measure regulation strategies. The continued use of these established scales allows researchers to build on prior theoretical bases. In line with the view of validity pertaining to contextualised results, complexity of the construct, and purpose of use of the results, the researcher needs to interpret results on regulation strategies in relation to the students’ study situation [9].

Scale scores should be interpreted in conjunction with other empirical data, such as course activities or other scale scores, thereby contributing to further construct validity.

Further refinement of the wording of questionnaire items is recommended in this translation, as medical students found some items hard to deal with. This work should retain content validity in adhering to the theoretical base and use reported dimensionality coefficients (*Hi),* and future content validation of the wordings with student groups, preferably from other disciplines as well. The vocational character of the participants’ study environment is currently marginally reflected in the strategy scales. Considering the contextual influence discussed by Richardson [10], more accurate results would perhaps be found in medical and other professional education if the scale items were adapted toward vocational aspects. However, as item adaptation would imply deviation from the original scales, and thus, the theoretical base, a better approach would be to add a vocational oriented scale along with the others. Generalizability of findings of this study beyond third-year students in a traditional Swedish medical curriculum is restricted by the moderate sample size and single discipline context. Data-gathering from the whole cohort, cross-sectionally over four course settings, contribute to the strength of the study, providing a broad base for validation of scale results.

## Conclusion

The regulation scales of the ILS measures individual study strategies relating to specific learning environments and, hence, to the culture in which they are used. These scales were developed in a non-medical education setting. Still, when used in a Swedish medical education setting, the scale structure is replicated, and internal consistencies are good. Thorough analysis of scale homogeneity identified areas where these scales can be further improved and possibly more tailored toward the professional aspects of medical education.

## Competing interests

The author declares no competing interests.

## Pre-publication history

The pre-publication history for this paper can be accessed here:

http://www.biomedcentral.com/1472-6920/12/76/prepub
